# Genetic analysis and elite tree selection of the main resin components of *slash pine*

**DOI:** 10.3389/fpls.2023.1079952

**Published:** 2023-02-01

**Authors:** Xianyin Ding, Yanjie Li, Yini Zhang, Shu Diao, Qifu Luan, Jingmin Jiang

**Affiliations:** ^1^ Research Institute of Subtropical Forestry, Chinese Academy of Forestry, Hangzhou, China; ^2^ Exotic Pine Cultivation Engineering Technology Research Center of National Forestry and Grassland Administration, Hangzhou, China

**Keywords:** slash pine, main resin components, genetic variation, correlation, genetic selection

## Abstract

Pine resin, as a natural material, has been widely used in food, pharmaceutical, and chemical industries. Slash pine (*Pinus elliottii* Engelm var. *elliottii*) is the primary tree species for resin tapping due to its high resin yield, low resin crystallization rate, and high turpentine content. Current researches focuse on the targeted improvement of several significant components to meet industrial needs rather than just resin yield. The objective of this study was to examine the genetic variation and correlation of genetic and phenotype for four main resin components (α pinene, β pinene, abietic acid, and levoprimaric acid) of 219 half-sib progenies from 59 families. The results showed that the levopimaric acid had the largest content (mean value = 21.63%), while the β pinene content had the largest variation coefficient (CV = 0.42). The α pinene content has the highest heritability (*h^2^* = 0.67), while levopimaric acid has the lowest heritability (*h^2^* = 0.51). There was a significant negative correlation between α pinene and the other three components and a significant positive correlation between β pinene and the two diterpenes. The family ranking and genetic gain suggested that it is possible to improve the contents of main resin components of slash pine through genetic breeding selection.

## Introduction

Pine resin is a mixture of terpenoids composed of volatile turpentine (monoterpenes and sesquiterpenes) and nonvolatile rosin (diterpene resin acids) (Diao et al., 2022). It is a renewable and eco-friendly biomass resource that plays an important role in the resistance of organisms to biotic and abiotic stress tolerance. In addition, pine resin has been widely used in the forest chemical industry (da Silva Rodrigues-Corrêa et al., 2013), such as in cosmetics, medicine, pesticides, spices, adhesives, and new biomass fuels (Luan et al., 2021). The reason for the functional diversity of rosin is the abundance of single components and combinations of different components (Lai et al., 2020). For example, α pinene and β pinene are well known as anti-inflammatory, analgesic (da Silva Rivas et al., 2012), inhibitors of breast cancer, and leukemia (Zhou et al., 2004), as well as play a crucial role in the fragrance and flavor industry (Allenspach and Steuer, 2021); abietic acid exerts *in vivo* anti-inflammatory activity and has partial ability to prevent the production of some inflammatory mediators (Fernandez et al., 2001); and pimaric-type acids, including pimaric acid, sandaracopimaric acid, isopimaric acid, and levopimaric acid, can be used to prepare anti-inflammatory and anticancer agents. A complete understanding of the genetic variation and correlation between different components of pine resin is essential for developing appropriate targeted breeding strategies.

Early researches on pines focused on the growth and wood traits, and it was not until 1940 that work on genetic improvement of resin traits in *Pinus palustris* Miller was first reported in America (Liefeld, 1940). Since the 1980s, studies to reveal the laws of genetic variation in pine resin yield and components have continued to emerge worldwide. For example, Austria selected the high-resin-yielding individuals of *Pinus nigra* (Rezzi et al., 2005); Portugal and Spain completed the high-resin-yielding breeding of *Pinus pinaster* (Rubini et al., 2021). China is a major producer and exporter of pine resin in the world, and domestic researchers have conducted many researches on its genetic improvement (Yi et al., 2021). The genetic variation in resin components of *Pinus massoniana* (Liu et al., 2016; Wu et al., 2019) and *Pinus kesiya* (Lai et al., 2020) has been quantified in recent years. The α pinene content in some individuals of *Pinus merkusii* is much higher than the other (Jantan et al., 2002), indicating that there is significant variation in the chemical components of resin between families and between individuals within families. These studies have revealed the genetic characteristics of different resin components, which can contribute to targeted breeding of various resin components.

Slash pine (*Pinus elliottii* Engelm var. *elliottii*), originally from the southeastern United States and widely planted in southern China in the 1930s, is the primary tree species for resin tapping because of its high resin yield, low resin crystallization rate, and high turpentine content (Nelson et al., 2013). In the 1980s, the United States carried out the first-generation selection of slash pine, and the resin yield increased by more than 60%-100%, and then established a high-resin-yielding seed orchard (McReynolds and Gansel, 1985). In China, the studies on genetic improvement of resin traits occurred later than that in the United States; thus far, a small number of high-resin-yielding seed orchards have been established (Zhang et al., 2016; Yi et al., 2021). Studies have shown that the characteristics of resin components in different geographical distributions of slash pine are basically the same, indicating its high heritability (Song et al., 1993). The genetic improvement effect of pine resin traits may be more significant, which can encourage researchers to select elite trees with multiple excellent traits.

To understand the quantitative genetics of the four main resin chemical components of slash pine, a study using 219 progeny trees of 59 families was conducted, and the research aims were as follows: (1) to examine and describe the four resin components of slash pine; (2) to evaluate the narrow-sense inheritance of the four resin components; (4) to determine the genetic and phenotype correlations within the four resin components; (3) to rank the families and select the elite families and individuals by the breeding value of the four resin components; and (5) to obtain the genetic gain of the four resin components at different selection intensities.

## Materials and methods

### Materials

All 59 half-sib families with 6 replications and 6 individual trees of slash pine were planted in a randomized complete block of a 2 m × 3 m spacing in the Yuhang region in Hangzhou, China (30°27′N, 119°49′E), established in March 1994. The specific experimental design has been detailed in Ding (Ding et al., 2022).

### Sample collection

Pine resin samples were collected in July 2021 from individuals whose diameter at breast height was close to the mean value of a family. A special plastic pipe with an aperture of 20 mm and volume of 15 ml was fixed at the drilling hole in the trunk to collect resin from the sunny side (Zhang et al., 2017). In total, we collected the resin from 219 representative individuals from 59 unrelated open-pollinated families (**Table S1**).

### Gas chromatography-mass spectrometry (GC-MS) analysis

Gas chromatography experiments were carried out with a GC 6890 gas chromatograph coupled with a Hewlett Packard GC 5975B mass spectrometer (Agilent 5975B, Santa Clara, CA, USA). The GC-MS operation was performed according to the procedure described by (Chen et al., 2020). To obtain the resin component content, 0.05 g resin was dissolved in 0.5 mL of ethyl alcohol containing 50 µL tetramethylammonium hydroxide. The GC column temperature conditions were as follows: the initial column temperature was 60°C, held for 2 min, increased at 8°C min^-1^ to 80°C, and reached a maximum of 280°C at a rate of 2°C per min for 5 min. The helium gas flow was set at 1 ml min^-1^. The temperature of the injector was 260°C, and the volume was 1 µL with a 1/50 split ratio. Mass spectra were recorded under electron impact ionization at an electron energy of 70 eV in the range from m/z 30 to 600 along with solvent delay for 3 min.

Resin compositions were identified by matching experimental fragmentation patterns in mass spectra with the NIST08 database through the data processing system of Agilent Chem Station and then comparing with the relevant literature. Monoterpene and sesquiterpene content were determined by isobutylbenzene content, and diterpene content was determined by heptadecanoic acid content. We calculated the resin component content by comparing their peak areas.

### Statistical analysis

Mixed linear model (LMM) with restricted maximum likelihood (REML) analysis was used for the estimation of genetic parameters. The model for a single-trait observation *y_i_* for a tree is shown in Equation (1):


(1) $$y_{i}\;=\;x_{i}m\;+\;b_{i\;}+\;f_{i\;}\;+\;e_{i\;}$$

where *x_i_* is a vector linking the fixed effects *m* to the observation *y_i_*, and *b_i_*, *f_i_*, and *e_i_* are the random block, family, and residual effects, respectively. Stacking those vectors for all trees produces model Equation (2):


(2) $$\text{y}\;=\;\text{Xm}\;+\;{\text{Z}}_{1}\text{b}+\;{\text{Z}}_{2}\text{f}\;+\;\text{e}$$

where y is a vector of total phenotypic observations, m is the vector of fixed effects (overall mean), and b, f, and e are vectors of bivariate random effects for block, family, and residual effects, respectively. X, Z1, and Z2 are the incidence matrices linking observations to the appropriate effects. We defined the vector of expected values (E) and dispersion matrices (Var) as:


(3) E[y]=Xm

(4) $$Var[\text{b}]={\text{Z}}_{1}⊗{\text{B}}_{0}$$

(5) $$Var[\text{f}]={\text{Z}}_{2}⊗{\text{F}}_{0}$$

(6) $$Var[\text{e}]=\text{Z}\;⊕{\text{R}}_{0}$$

and


(7) $${\text{B}}_{0}=\left[\begin{array}{cc} \sigma_{b1}^{2} & \sigma_{b1b2}\\ \sigma_{b2b1} & \sigma_{b2}^{2} \end{array}\right]$$

(8) $${\text{F}}_{0}=\left[\begin{array}{cc} \sigma_{f1}^{2} & \sigma_{f1f2}\\ \sigma_{f2f1} & \sigma_{f2}^{2} \end{array}\right]$$

(9) $${\text{R}}_{0}=\left[\begin{array}{cc} \sigma_{e1}^{2} & \sigma_{e1e2}\\ \sigma_{e2e1} & \sigma_{e2}^{2} \end{array}\right]$$

where ⊗ and ⊕ are the direct product and direct sum operations, respectively; $\sigma_{bi}^{2}$ , $\sigma_{f1}^{2}$ , and $\sigma_{e1}^{2}$ represent the block, family, and residual variances for trait *i*, respectively; and *σ_bibj_* , *σ_fifj_*, and *σ_eiej_* are the covariances of block, family, and residual between traits *i* and *j*.

The variance components from the model were used to calculate the narrow sense heritability (*h^2^*):


(10) $$h_{i}^{2}=\frac{4\sigma_{f_{i}}^{2}}{\sigma_{f_{i}}^{2}+\sigma_{b_{i}}^{2}+\sigma_{e_{i}}^{2}}$$

The genetic correlations (*r*_*g*_*ij*__ ) between trait *i* and trait *j* were calculated as:


(11) $$r_{g_{ij}}=\frac{\sigma_{f_{ij}}}{\sqrt{\sigma_{f_{i}}^{2}+\sigma_{f_{j}}^{2}}}$$

and phenotypic correlation ( *r*_*p*_*ij*__ ):


(12) $$r_{p_{ij}}=\frac{\sigma_{f_{ij}}\;+\;\sigma_{e_{ij}}}{\sqrt{\left(\sigma_{f_{i}}^{2}+\sigma_{e_{i}}^{2}\right)\left(\sigma_{f_{j}}^{2}+\sigma_{e_{j}}^{2}\right)}}$$

where *σ*_*f*_*ij*__ is the estimated family covariance between trait *i* and trait *j* and $\sigma_{f_{i}}^{2}$ and $\sigma_{f_{j}}^{2}$ are the estimated family variances for trait *i* and trait *j*.

The breeding value (BV) of each tree is the family random effect value of the LMMs, and the realized genetic gain (Δ*G_R_*) was computed by subtracting the mean BVs of the selected top ratio trait from its total mean.

The statistics were calculated by the “mmer” and “h2.fun” functions of the “sommer” package (Covarrubias-Pazaran, 2016), and the figures were plotted by the “ggplot2” package (Gómez-Rubio, 2017). All analyses were performed in the R environment (Team, 2013).

## Results

### Variation in resin components of slash pine

**Figure 1:**
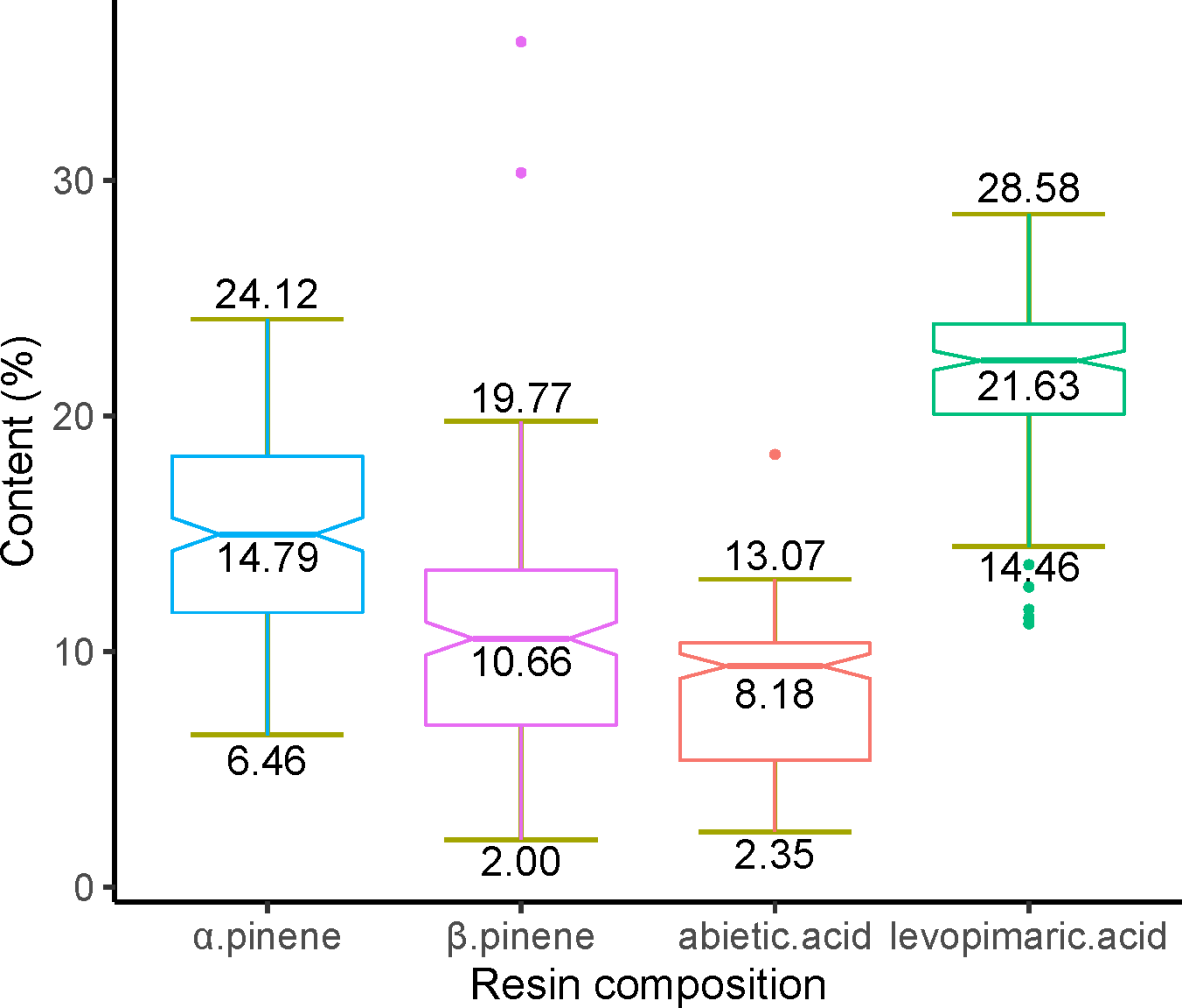
Variation in the α pinene, β pinene, abietic acid and levoprimaric acid contents. The minimum, maximum, and average values of each pine resin component content in the slash pine population were marked at the bottom, top, and middle of each boxplot, respectively.

In total, we obtained 10 monoterpenes, 3 sesquiterpenes, and 18 diterpenes (**Table S2**). We then selected four components believed to have broad industrial utility for subsequent analysis, including two monoterpenes (α pinene and β pinene) and two diterpenes (abietic acid and levoprimaric acid). The contents variation of the four compositions have a reasonable range (2.00% to 24.12% in monoterpenes and 2.35% to 28.58% in diterpenes) (**Figure 1**). Among monoterpenes, α pinene has the highest content (mean value = 14.79%), while levopimaric acid has the highest content in diterpenes (mean value = 21.63%). The β pinene has the largest content variation with a variable coefficient (CV) of 0.42, followed by abietic acid (CV = 0.37), α pinene (CV= 0.29) and levopimaric acid (CV = 0.15).

**Figure 2:**
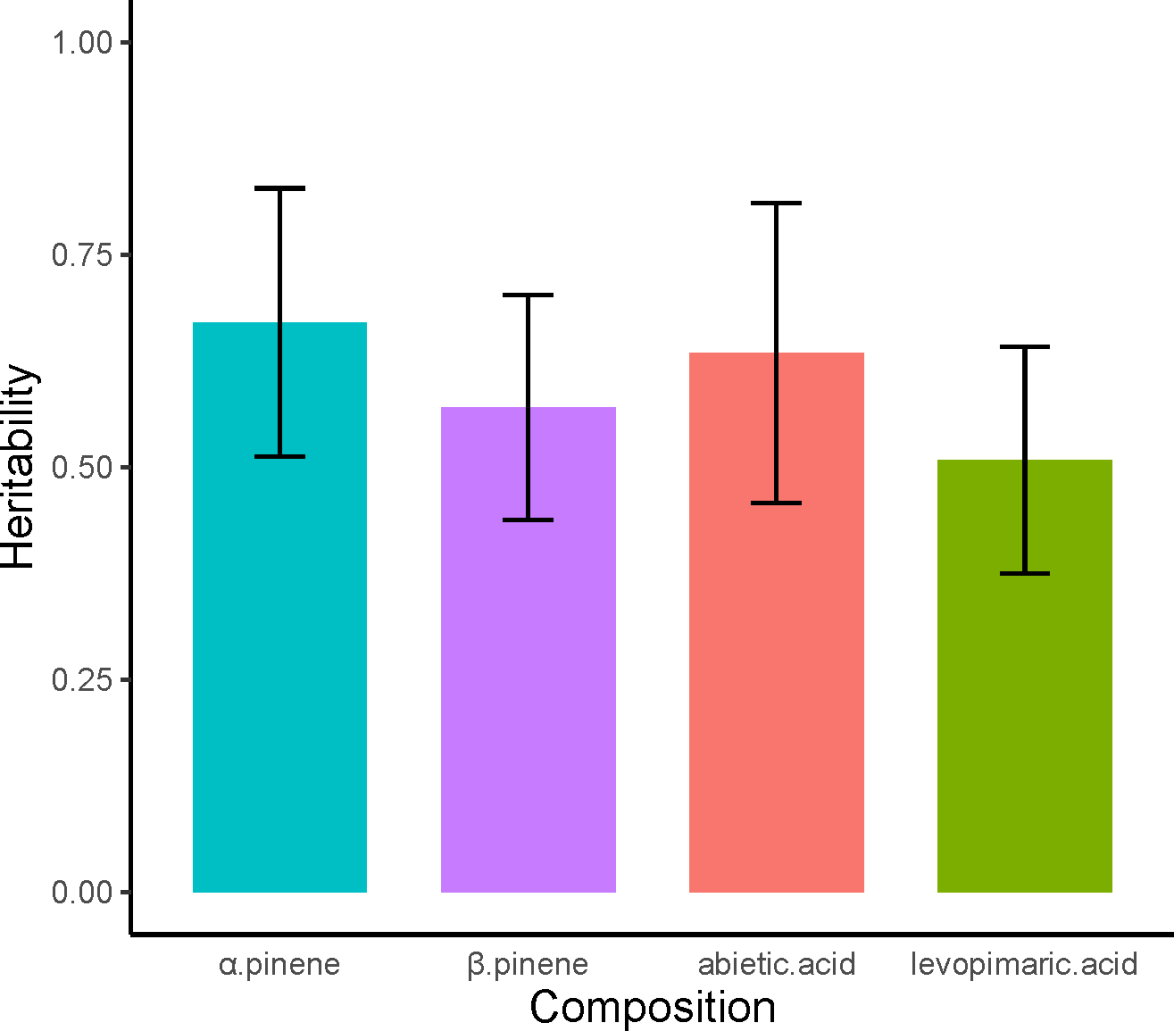
Heritability of α pinene, β pinene, abietic acid and levoprimaric acid. The red line at the top of each column shows the margin of standard error for heritability.

The estimated narrow-sense heritabilities (*h^2^*) of the four resin components were 0.67, 0.57, 0.63, and 0.51, respectively (**Figure 2**). Overall, the heritability of the four resin components was high and the monoterpenes had higher heritability than diterpenes. The *h^2^* of α pinene was the highest, while the levopimaric acid has the lowest *h^2^*. Additionally, the margin of system error for *h^2^* is small, between plus or minus 0.13 to 0.17. This indicated that the traits of resin components of slash pine were not greatly affected by environmental factors but were mainly controlled by genetics.

**Figure 3:**
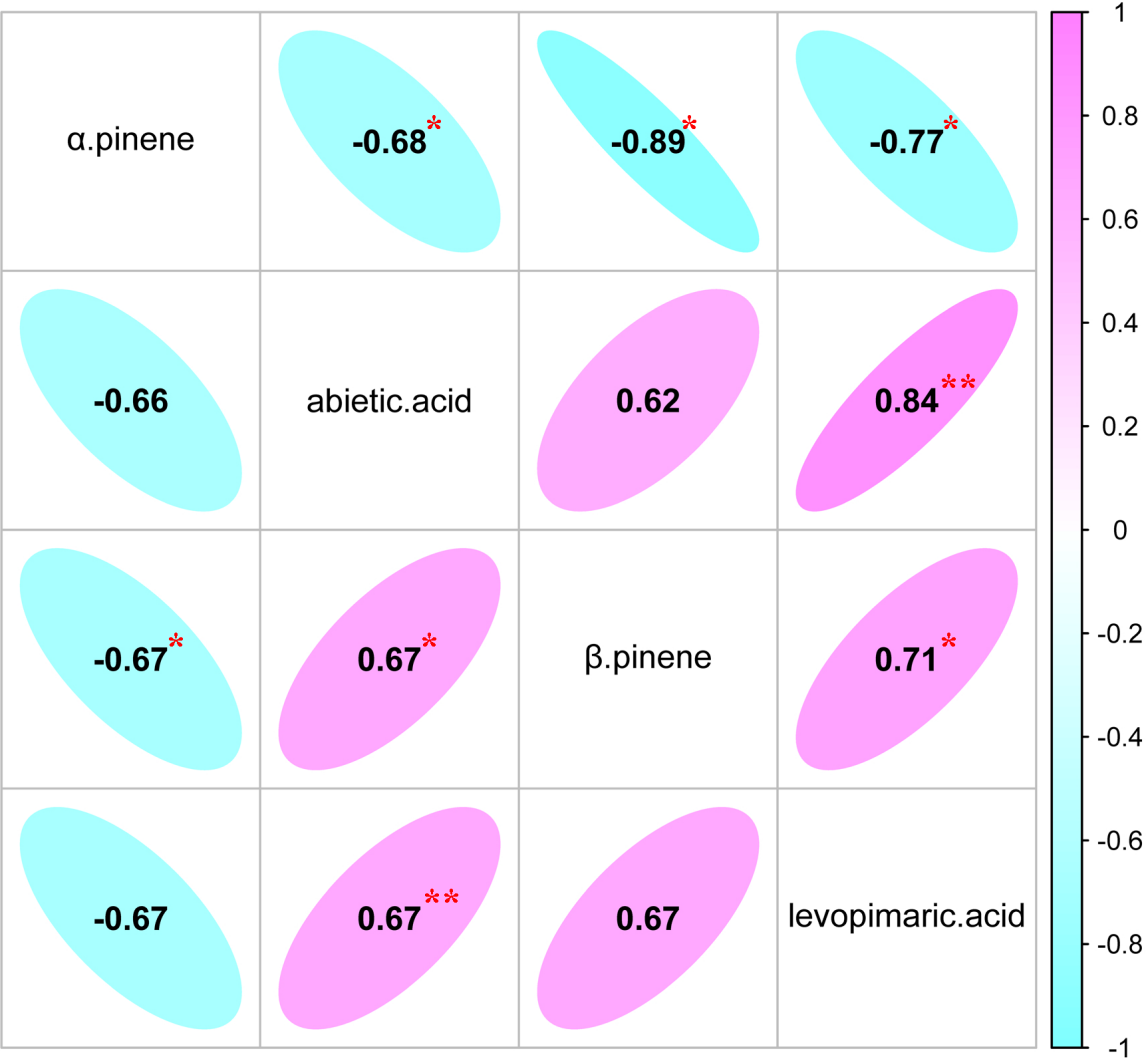
Estimates of genetic correlation (below the diagonal) and phenotypic correlation (above the diagonal) of different resin components.

The genetic (*r_g_*) and phenotypic correlation (*r_p_*) analyses of the four resin components of slash pine are shown in **Figure 3**. The strongest negative genetic correlation (*r_g_* = -0.67) and phenotypic correlation (*r_p_* = -0.89) were found between the two monoterpenes (α pinene and β pinene). The β pinene and abietic acid had the weakest genetic correlation (*r_g_* = 0.62) and phenotypic correlation (*r_p_* = 0.67). Interestingly, the genetic correlation (*r_g_* = 0.67) and phenotypic correlation (*r_p_* = 0.84) between the two diterpenes (abietic acid and levoprimaric acid) were stronger than those between monoterpenes and diterpenes.

### Ranking of breeding value and selection of excellent families with different resin components

**Figure 4:**
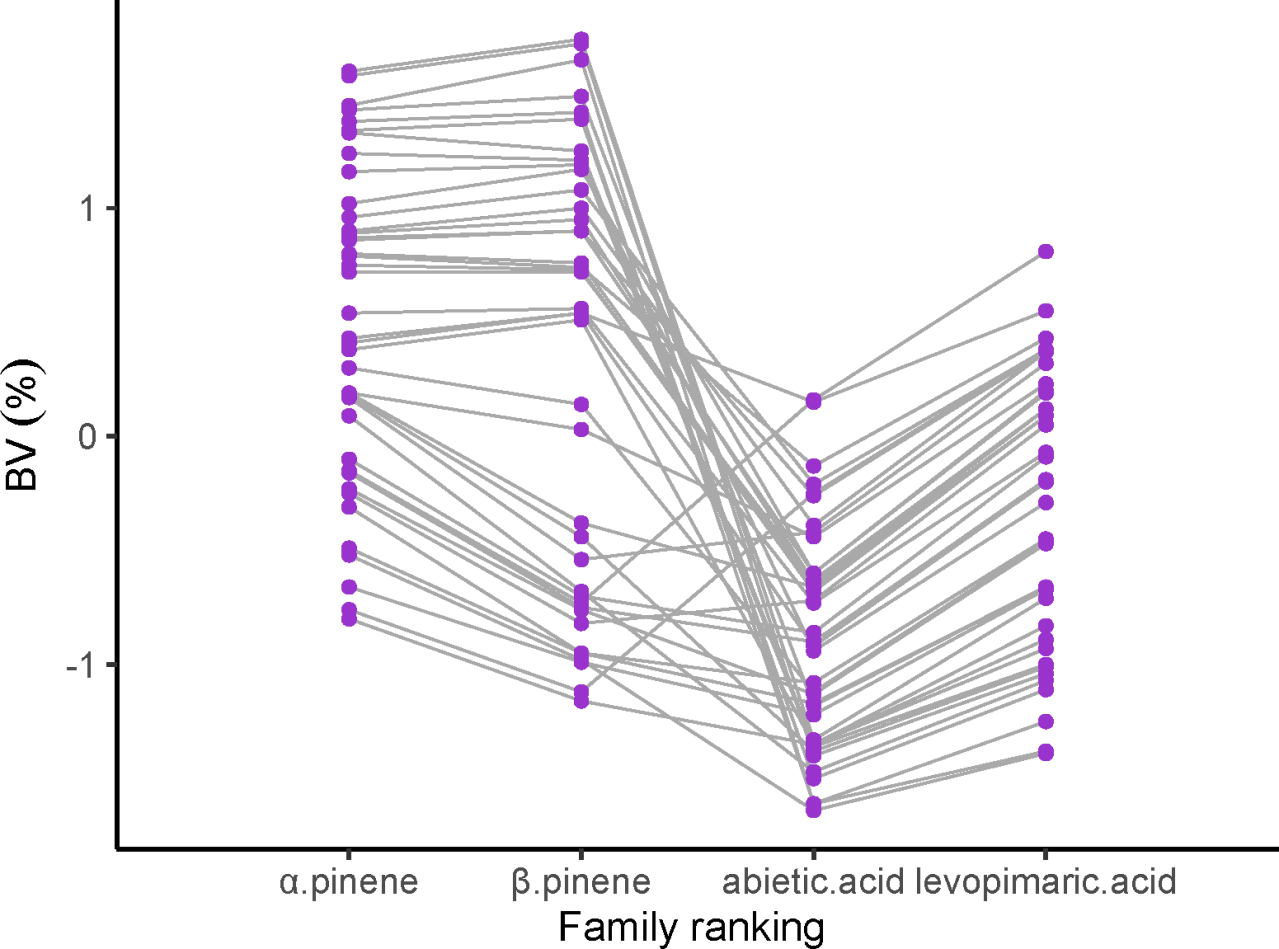
Fifty-nine families rankings for breeding value (BV) of the α pinene, β pinene, abietic acid and levoprimaric acid in slash pine at age 29. Each purple dot and gray line represent one family.

We ranked the family performance of slash pine by a breeding value (BV) of α pinene, β pinene, abietic acid and levoprimaric acid in **Figure 4**. Apparently, α pinene had a significantly consistent family ranking with β pinene, while a similar result was also found between abietic acid and levoprimaric acid. Unfortunately, β pinene and abietic acid did not show a highly consistent family ranking, indicating a worse correlation between the monoterpenes and the diterpenes, which was consistent with the conclusion of **Figure 3**. However, it was still possible to select traits by families according to certain purposes.

**Figure 5:**
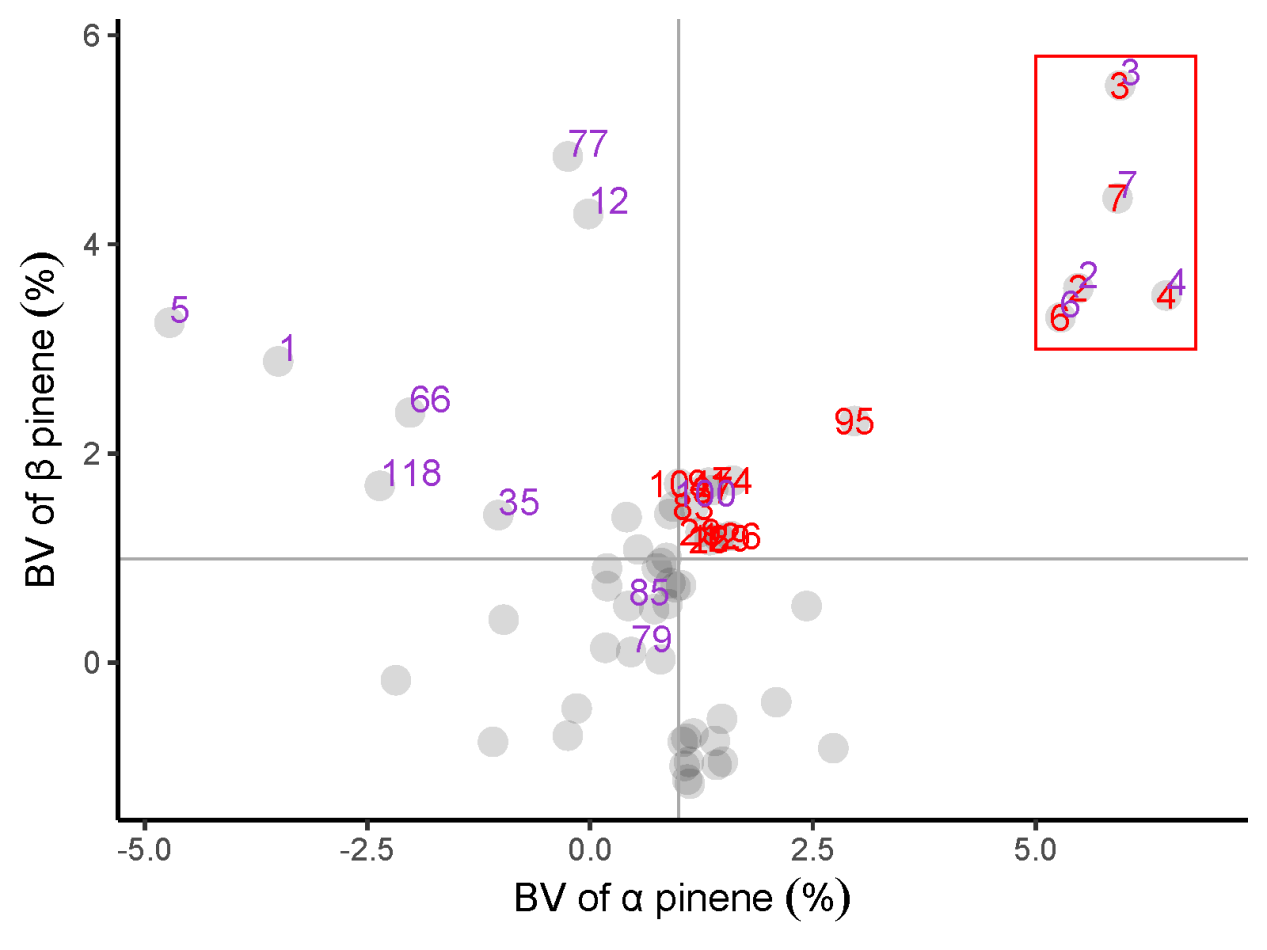
Relationship between α pinene, β pinene, abietic acid and levoprimaric acid BV of the slash pine family. Each large gray dot represents one family. Red label: BV of α pinene, β pinene above the mean value; Purple label: BV of abietic acid and levoprimaric acid above the mean value. A family with two color tags in the red box was considered to perform excellently. The mean BV of α pinene and β pinene are displayed as a gray solid line.

The relationship between α pinene, β pinene, abietic acid and levoprimaric acid traits is displayed in **Figure 5**. There were 15 families with BVs above the mean value of α pinene and β pinene that were marked in red. In addition, 15 families were identified that involved a BV above the mean of abietic acid and levoprimaric acid, which were labeled as purple. Fortunately, there are 5 families, including 2, 3, 4, 6 and 7, whose BVs are above the mean of all four resin components that deserve to be selected.

### Realizing a high genetic gain of four resin components

**Figure 6:**
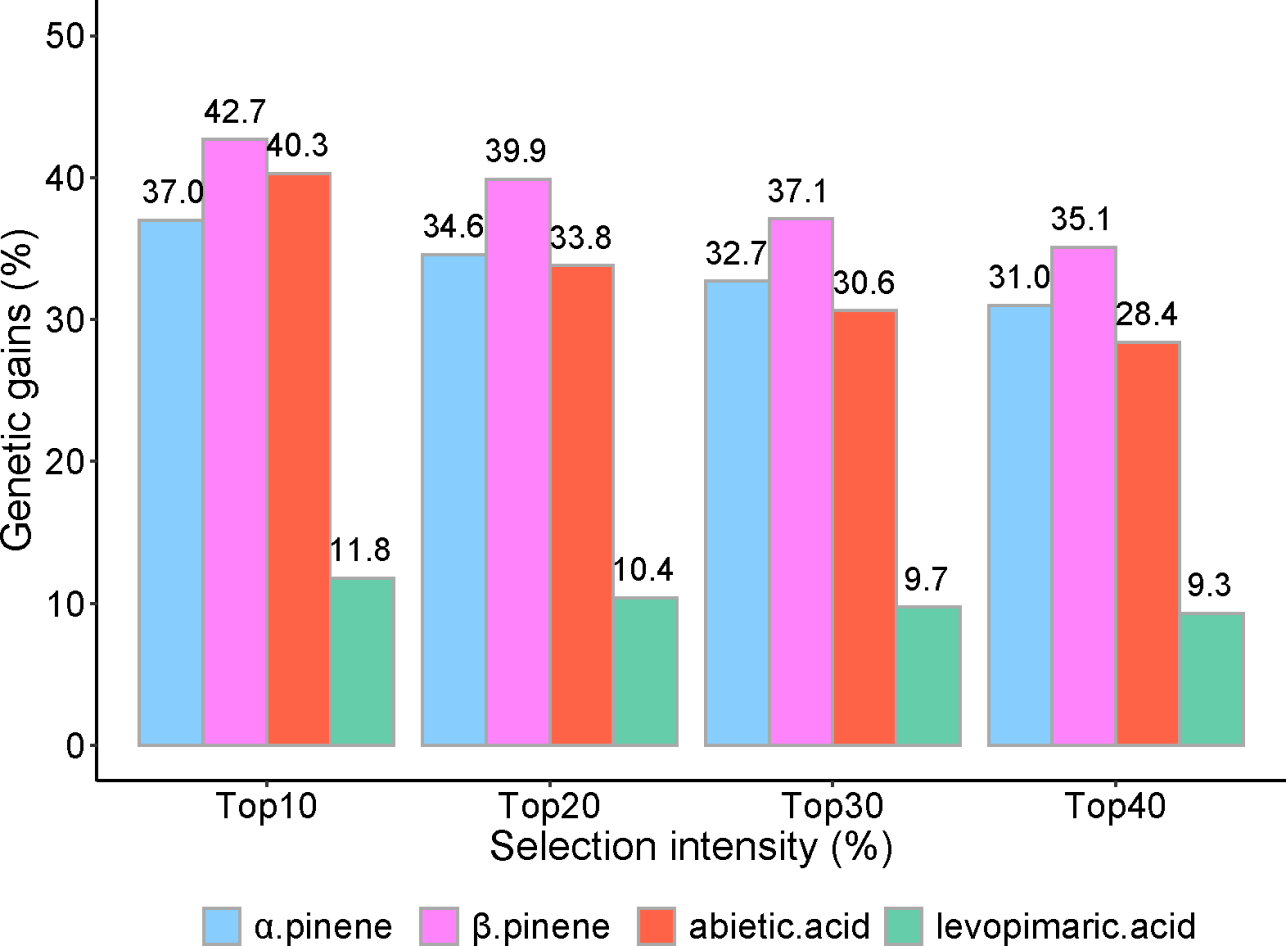
Realized genetic gains of α pinene, β pinene, abietic acid and levoprimaric acid at age 29 for slash pine. Selection intensity ranged from 10% to 40% of the 59 families; genetic gains were calculated as an improved percentage.

The ΔG% (improved percentage of the genetic gain) was calculated with the top 10% to 40% samples from 59 families for each resin component (**Figure 6**). On the whole, the top 10% of families could produce the highest yield of pine resin among other selection intensities. Except for the top 10%, there was little difference in the performance of the other three intensities. The top 10% yielded nearly 1.5 times as much as the top 40% selection intensity in abietic acid but not significantly in α pinene, β pinene, and levoprimaric acid.

The largest average ΔG% was observed in β pinene, where the top 10% to 40% families yielded 42.7%, 39.9%, 37.1, and 35.1%, respectively, followed by α pinene, abietic acid, and levoprimaric acid, with estimated genetic gains ranging from 37.0% to 31.0%, 40.3% to 28.4%, and 11.8% to 9.3%, respectively.

## Discussion

### The abundance of genetic variation determines the depth of genetic improvement

In this study, we first assessed the variation and heritability of four relatively important resin components in the slash pine population. The results showed that the content of α pinene is approximately 1.4 times higher than that of β pinene, which was similar to the study conducted by Zhang (Zhang et al., 2016) and Lai (Lai et al., 2020). However, the content of α pinene accounted for more than 90% of the total monoterpenes in Masson pine (Liu et al., 2016). The content of levopimaric acid was the highest, accounting for 21.63% of the total content on average, followed by abietic acid, accounting for 8.12%, which were, respectively higher and lower than the results of previous similar studies (Zhang et al., 2016; Lai et al., 2020). These differences may be attributed to differences in environmental and climatic conditions. In addition, turpentine is volatile, and errors in resin collection and measurement must be accounted for (Luan et al., 2022).

Heritability is an important population parameter that can help understand the genetic architecture of complex traits (Lee et al., 2011). In this study, the four resin components were under strong genetic control, which was higher than the results estimated by Lai (Lai et al., 2020). A similar result that heritability of α pinene is higher than β pinene was also found both in slash pine (Zhang et al., 2016) and Masson pine (Liu et al., 2016). An estimate of the heritability of a trait is specific to population, species, and environment, and it may change over time (Li et al., 2020). High heritability will drive the process of genetic improvement of these four important resin components of slash pine. The heritability estimated in this study would be significant for our slash pine population and has some reference value for other studies.

### Correlation analysis can direct the development of targeted breeding strategies

Understanding the genetic and phenotypic correlation among traits contributes to researchers developing breeding strategies more effectively (Lee et al., 2002; Li et al., 2020). The correlation analysis of genetic and phenotypic of the four main resin components found that two monoterpenes (α pinene and β pinene) exhibited a strongly negative correlation, and α pinene and β pinene had negative and positive correlations with the two diterpenes (levopimaric acid and abietic acid), respectively, which agrees with the results of previous reports (Lei et al., 2015; Zhang et al., 2016; Lai et al., 2020). It is believed that α pinene and β pinene may be controlled by the same pair of alleles, and the syntheses of these two components are negatively correlated (Li et al., 2012). Alternatively, they are controlled by different genes, and their synthesis signals suppress each other (Hall et al., 2013; Diao et al., 2022). There was a strongly positive genetic and phenotypic correlation between β pinene and two diterpenes with high content in this study. Previous studies have also shown that β pinene is significantly related with resin yield traits, which highlights the importance of β pinene in high yield of pine resin breeding (Karanikas et al., 2010; De Lima et al., 2016; Neis et al., 2019).

### Elite family selection and genetic gain statistics

Simultaneous selection of several important monoterpenes and diterpenes in resin is desirable for breeders in a long-term breeding program. Our family breeding value (BV) study of four important slash pine resin components provided the possibility for their joint selection. This method has been successfully applied to the combined selection of slash pine growth and wood traits (Li et al., 2020), as well as the combined selection of leaf color parameters and chlorophyll of *Sassafras tzumu* (Li et al., 2019). In this study, we selected 5 families, 2, 3, 4, 6, and 7, all of which contained high levels of α pinene, β pinene, abietic acid and levoprimaric acid, providing an important reference for the subsequent establishment of high-resin-yielding slash pine seed orchards.

Genetic gain can characterize the effect of direct or indirect breeding selection. Stronger genetic selection ratios tend to lead to higher genetic gain (Li et al., 2018; Zhang et al., 2018), and the same patterns were also reflected in this study. The β pinene possesses the highest genetic gain, which means that our high resin yield breeding for slash pine is effective.

## Conclusions

Resin is an important nontimber secondary forest product. In this study, a total of 219 individuals from 59 slash pine families were used for resin tapping. Then, we used GC-MS to characterize and quantify different resin components. Different from the usual selection of resin yield and mixture of turpentine or rosin in previous studies, we selected four components considered to be more important, including two kinds of turpentine (α pinene and β pinene) and two kinds of resin acids (levopimaric acid and abietic acid). Industrial production often requires some important chemical components rather than the entire resin. To this end, we first analyzed the genetic variation in the contents of the four resin components. The results suggested an abundant variation and a strong genetic control in resin components of slash pine breeding population. The β pinene has the largest variation coefficient, and the α pinene has the highest heritability. Then, correlation analysis revealed a law consistent with the other results found in previous studies on slash pine resin. Moreover, we ranked the families by breeding value and selected 5 families, including 2, 3, 4, 6 and 7, whose BVs were above the mean of all four resin components. Finally, we found that the largest average ΔG% was observed in β pinene, while the lowest was observed in levopimaric acid at all selection intensities. These results help us to gain a deeper understanding of the genetic basis of resin traits of slash pine and facilitate its genetic improvement.

## Data availability statement

The original contributions presented in the study are included in the article/**Supplementary Material**. Further inquiries can be directed to the corresponding author.

## Author contributions

Conceptualization: QL and XD. Data curation: QL and JJ. Formal analysis: XD. Funding acquisition: QL. Investigation: QL, XD, and YZ. Methodology: QL and XD. Project administration: QL. Resources: QL and JJ. Software: XD. Supervision: JJ, QL, YL, and SD. Validation: QL. Visualization: XD. Writing – original draft: XD. Writing – review & editing: QL, YL, SD, and XD. All authors contributed to the article and approved the submitted version.

## Supplementary material

Table S1 Sample information.

Table S2 Resin components of 240 accessions of *Pinus elliottii*.

Supplementary Data Sheet 1–6 S1-S6 Data. The source data for Fig. 1 to Fig. 6.
